# Small Molecule Subgraph Detector (SMSD) toolkit

**DOI:** 10.1186/1758-2946-1-12

**Published:** 2009-08-10

**Authors:** Syed Asad Rahman, Matthew Bashton, Gemma L Holliday, Rainer Schrader, Janet M Thornton

**Affiliations:** 1 EMBL-European Bioinformatics Institute, Wellcome Trust Genome Campus, Hinxton, Cambridge, CB10 1SD, UK; 2 Zentrum für Angewandte Informatik (ZAIK), Universität zu Köln, Weyertal 80, D-50931 Köln, Germany

## Abstract

**Background:**

Finding one small molecule (query) in a large target library is a challenging task in computational chemistry. Although several heuristic approaches are available using fragment-based chemical similarity searches, they fail to identify exact atom-bond equivalence between the query and target molecules and thus cannot be applied to complex chemical similarity searches, such as searching a complete or partial metabolic pathway.

In this paper we present a new Maximum Common Subgraph (MCS) tool: SMSD (**S**mall **M**olecule **S**ubgraph **D**etector) to overcome the issues with current heuristic approaches to small molecule similarity searches. The MCS search implemented in SMSD incorporates chemical knowledge (atom type match with bond sensitive and insensitive information) while searching molecular similarity. We also propose a novel method by which solutions obtained by each MCS run can be ranked using chemical filters such as stereochemistry, bond energy, etc.

**Results:**

In order to benchmark and test the tool, we performed a 50,000 pair-wise comparison between KEGG ligands and PDB HET Group atoms. In both cases the SMSD was shown to be more efficient than the widely used MCS module implemented in the Chemistry Development Kit (CDK) in generating MCS solutions from our test cases.

**Conclusion:**

Presently this tool can be applied to various areas of bioinformatics and chemo-informatics for finding exhaustive MCS matches. For example, it can be used to analyse metabolic networks by mapping the atoms between reactants and products involved in reactions. It can also be used to detect the MCS/substructure searches in small molecules reported by metabolome experiments, as well as in the screening of drug-like compounds with similar substructures.

Thus, we present a robust tool that can be used for multiple applications, including the discovery of new drug molecules. This tool is freely available on http://www.ebi.ac.uk/thornton-srv/software/SMSD/

## Background

The chemical similarity between two molecules, either at the sub- or superstructure level [1], and clustering of similar molecules [2,3] are widely used to measure the diversity of the chemical space [4]. These methods are of vital importance, as they can be applied towards discovering new drug-like molecules [5-8]. The similarity between the sub- & superstructures can be measured by mathematical coefficients such as the Tanimoto similarity or Euclidean distance [9]. One of the most frequently used similarity search approaches is based on "structural descriptors" [1] and to date these have proved to be the most effective methods for measuring chemical similarity. Newer methods such as the Maximum Common Subgraph (MCS) have more recently come to the fore as they overcome nearly all of the challenges posed by fragment or descriptor based similarity searches [10]. The MCS methods benefit from much improved sensitivity as they can find atom-atom or atom-bond equivalence between query and target molecules [11]. It then becomes possible to follow a query substructure or moieties along metabolic pathways or reaction schemes.

One of the main issues with using the MCS is that it is NP-complete, i.e. no method can guarantee a perfect solution in polynomial time [12]. Many MCS algorithms have been proposed to overcome the complexity involved in finding chemically relevant substructures sharing the same pattern. The most widely used algorithm for the MCS problem of molecular graphs is the one designed by Bron and Kerbosch [13], which enumerates all cliques of the compatibility graph [14]. It applies a branch and bound technique to prune the search tree, which makes it very efficient. Some of these algorithms use heuristic approaches for arriving at non-optimal solutions [15-18] while others give optimal solutions [19]. Most of these algorithms find the MCS by converting a molecular association graph into a clique detection problem. An alternative MCS method has been proposed, without constructing an association graph, based on the backtracking algorithm [20,21]. Cao et al. [22] proposed an MCS algorithm based on the backtracking VF algorithm [23,24], with some heuristics for searching and predicting drug-like compounds. While a few of these algorithms [25]; Akutsu 2004 [16] are based on dynamic programming, others ([26] use heuristic algorithms to find subgraph isomorphism. Some of the above mentioned MCS algorithms utilize the labels of the vertices (atoms), the induced edges (bonds), and other important structural feature constraints. Interestingly almost all of them lack chemical knowledge to rank the MCS solutions based on information such as bond energies.

In this paper we present a chemically sensitive and robust tool, which uses a combination of various graph-matching algorithms for finding the MCS between small molecules.

This new tool can generate bond sensitive and bond insensitive MCS and it ranks the solution(s) according to minimal fragments, bond breaking energy and stereochemical matches. It can work with explicit hydrogen(s) though it performs faster when the hydrogen(s) are implicit (*i.e*. hydrogen(s) are only considered to be present where explicitly defined by the input molecule's structure). The tool's performance is benchmarked on a small virtual screening test case between KEGG [27] and PDB [28] molecules (50,000 pair-wise comparisons). It is difficult to compare the MCS algorithms implemented in various chemical tools, since most of them are commercial. Hence we have chosen the widely used MCS module implemented in an open source chemo-informatics library: the MCS algorithm in the Chemistry Development Kit (CDK) [29]. The new tool uses the CDK where efficient, but also provides solutions in cases where the MCS routine in the CDK fails to provide a solution.

The design principles of graph matching algorithms are all closely related to the kind of problem they address. Since the specificity and sensitivity of the chemical similarity/distance searches are a function of time and space, heuristic techniques [17] have evolved to map two molecules.

### Chemical Graph Theory and MCS

Graph theory studies pair-wise relationships between objects. A molecular graph *G *= (*V*, *E*, *l*) consists of a set of vertices *V*(*G*) (i.e. atoms in a molecule), a set of edges *E*(*G*) (i.e. the bonds in a molecule) and *l *is a function that maps the union of *V *and *E *to natural numbers (maps atoms and bonds to their types). Molecular graphs are assumed to be simple, undirected graphs. A molecular graph *G*_*m *_consists of a set of vertices *V*(*G*_*m*_) and a set of edges *E*(*G*_*m*_). The vertices in *G *are connected by an edge if there exists an edge (*v*_*i*_, *v*_*j*_) ∈ *E*(*G*) connecting the vertices *v*_*i *_and *v*_*j *_in *G *such that *v*_*i*_, *v*_*j *_∈ *V*(*G*).

Two graphs *G*_*q *_(query) and *G*_*t *_(target) are said to be *isomorphic *if there is a one-to-one correspondence (mapping) *f *: *V*(*G*_*q*_) → *V*(*G*_*t*_) between their vertex sets which preserves the adjacency of vertices (i.e. two vertices *u *and *v *from *G*_*q *_are adjacent if and only if *f *(*u*) and *f *(*v*) are adjacent in (*G*_*t*_). The mapping itself is called an *isomorphism*. Two labeled graphs *G*_*q *_and *G*_*t *_are isomorphic if there is an isomorphism *f *between them preserving the labels i.e. *l*(*v*) = *l*(*f *(*v*)) for all *v *∈ *V*(*G*_*q *_and *l*(*u*, *v*) = *l*(*f *(*u*), *f *(*v*)) for all edges *u*, *v *∈ *E*(*G*_*q*_).

A *clique *(ω) in a molecular *G*_*m *_can be defined as a subset of vertices such that each pair of vertices is connected by an edge in the graph *G*_*m*_. We call a subgraph  ⊆ *G*_*m *_*complete *if *u*, *v *∈ *E *for all *u*, *v *∈ *G*(*V'*). The subgraph  is maximal if it is not a subgraph of a larger subgraph. A maximum clique is the largest maximal clique induced in *G*_*m*_.

A maximal clique ω(*G*_*m*_) in a graph *G*_*m *_is a clique of a graph that is not a subgraph of a larger clique in *G*_*m *_possessing these criteria (*i.e*. bonds, atom types and their connectivity are preserved), hence this subgraph is referred to as a *Maximum Common Subgraph *(MCS).

**Figure 1:**
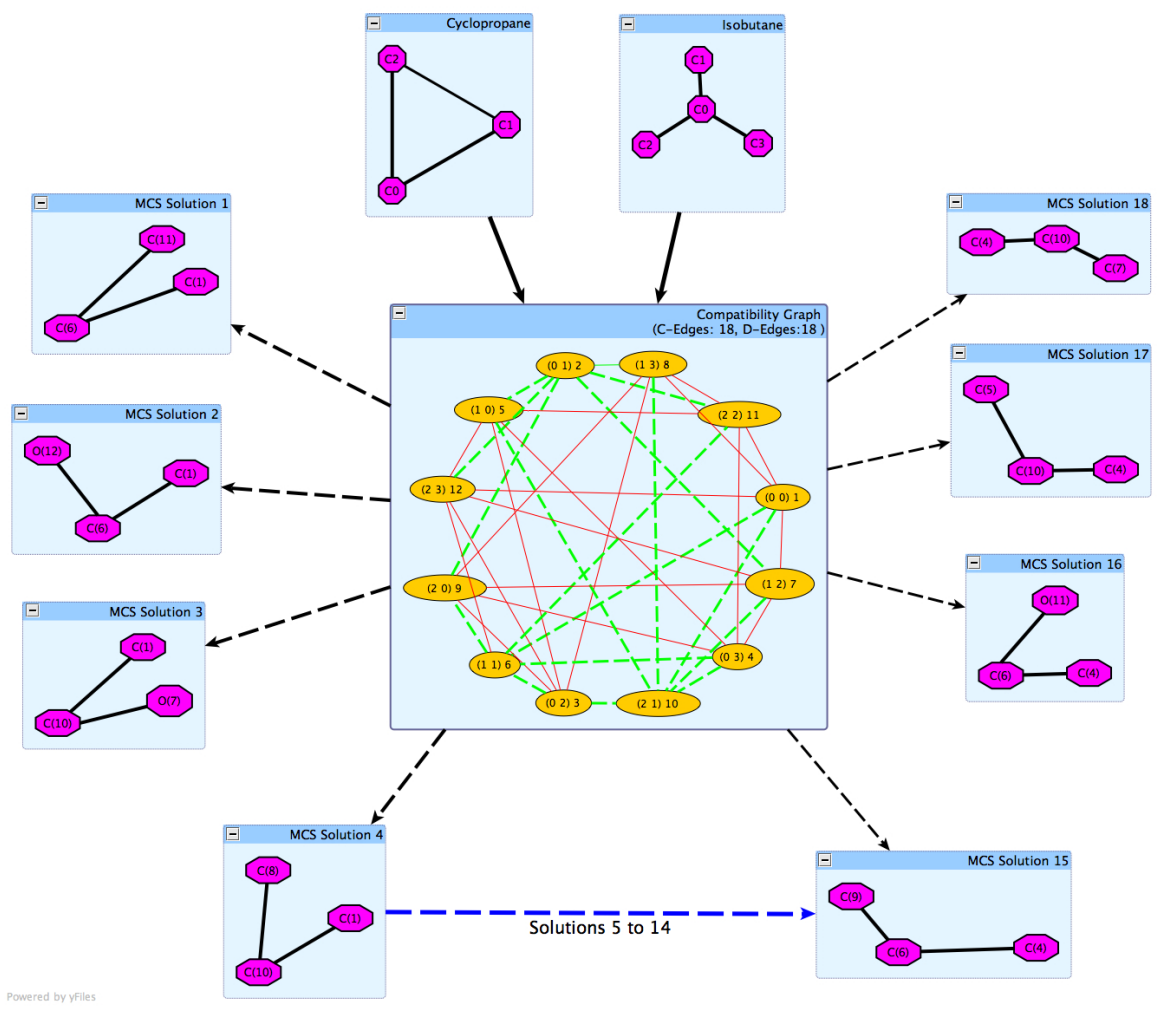
**The compatibility graph between Isobutane and Cyclopropane will generate a compatibility graph with 36 edges and 12 vertices**. There are 18 c-edges (green dotted lines) and non *c-edges *(red lines). This will lead to 18 MCS solutions, each of size 3.

The modular product of two graphs *G*_*q *_(query) and *G*_*t *_(target) is defined on the Cartesian product *V*(*G*_*q*_) ⊗ *V*(*G*_*t*_) with any two vertices *u*_*q*_, *v*_*q *_and *u*_*t*_, *v*_*t *_being adjacent in the modular graph. The modular product will be denoted by *G*_*q *_⊗ *G*_*t*_. In the case of molecular graphs (labelled graphs), the modular product of two graphs can be further restricted by constraints such as the vertex and edge labels satisfying certain compatibility criterion. Hence, in the case of molecular graphs, the modular product graph is often called compatibility graph (Figure 1).

## Implementation

**Figure 2:**
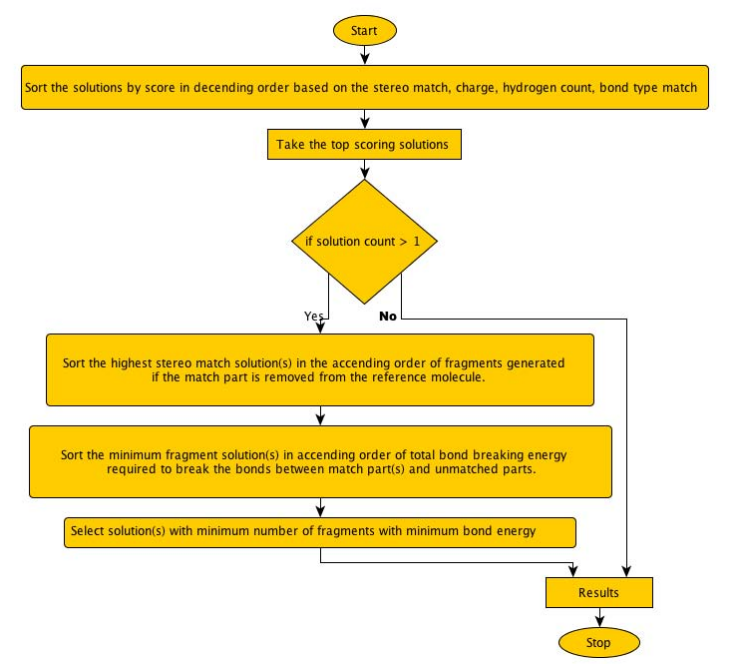
**Flowchart describing the post-filtering step in the SMSD algorithm**.

**Figure 3:**
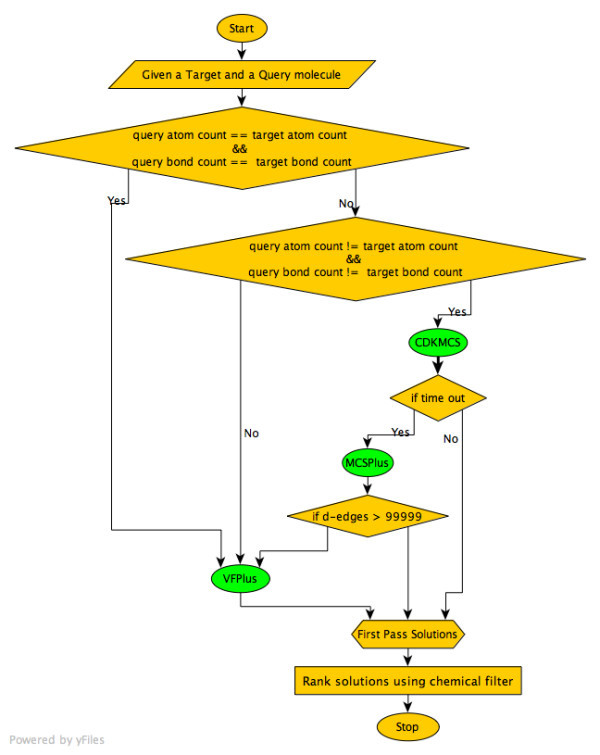
**Flowchart outlining the methodology of the Maximum Common Subgraph (MCS) algorithm used by SMSD**.

We propose a new Java based tool called Small Molecule Subgraph Detector (SMSD) to find the MCS in small molecules. The SMSD is distinguished from previous algorithms in that it uses a combination of various algorithms (summarised in appendix 1) to find the MCS and filters the results in a manner that is chemically relevant (Figure 2). In the following section we describe the underlying algorithm and the subroutines (Figure 3), which take account of the chemistry in the SMSD for the MCS. The SMSD calculates the MCS between two molecules by combining the power of the VF+ Lib, the MCS+, and the CDK based MCS algorithm. These algorithms are used automatically on a case-by-case basis, which is dependent on the molecules under consideration for the MCS search.

### Revisiting the MCS routine in the CDK (Chemistry Development Kit)

The MCS algorithm as coded in the CDK [30] (CDKMCS) is based upon the common pattern recognition concept and the principle of Maximum Common Induced Subgraph (MCIS) [19]. In that approach, a given molecular graph *G*_*m *_is reduced to a MCIS graph *G'*. This algorithm is designed in such a way that it selects the common features between two graphs using bit strings. Thus, a compatibility graph between two molecules can be traversed in a much faster way. In order to traverse the edges on the graph it uses an approach similar to the Bron and Kerbosch [13] algorithm.

The disadvantages of the MCS algorithm in the CDK are:

a) It may treat two chemically non-identical molecules as identical because it works on the MCIS principle, e.g. cyclopropane (CAS: 75-19-4) and isobutane (CAS: 75-28-5).

b) The runtime is high if two graphs are large with few dissimilar edges e.g. between aquacob(III)alamin (CAS: 13422-52-1) and ferroheme (CAS: 14875-96-8).

These challenges were resolved in the SMSD, by first using the atom and bond count filter to discriminate between two dissimilar structures (which the CDKMCS might consider similar) before performing the MCS search, and secondly, by using the VF+ Lib and MCS+ method developed herein.

### MCS+

The MCS+ method has been designed using a combination of a compatibility graph based clique detection algorithm and a backtracking algorithm. Koch [31] presented an efficient algorithm called *c-cliques *based on the MCES (or maximum overlapping set (MOS)) concept that identifies cliques by pruning a given graph on the basis of certain constraints (*c-edges *and *d-edges*) before using the Bron Kerbosch algorithm to find the MCS. Leber [32] used this algorithm to find MCS between reactants and products in a reaction. The drawback to this algorithm is that it is not guaranteed to give the optimal solution (maximum clique), *i.e*. the *c-cliques *reported by this method might not contain the complete maximum match. Hence Leber [32] used McGregor's backtracking algorithm to further extend the clique when possible. Thus, the resulting clique will contain the complete maximum match. The performance of the MCS+ algorithm is robust (in terms of time) especially in cases where two graphs are not identical as the unconnected/dissimilar parts are restricted in the pre-processing module (*c-edges *&*d-edges*).

In the Koch approach, an edge in the compatibility graph is designated a c-edge if two adjacent vertices in the graphs *G*_*q *_and *G*_*t *_are connected and they share similar properties. In our tool, these properties can be described as a similar bond type. However, if an adjacent edge in the graph is not connected then it is termed as a *d-edge*. As the *d-edges *represent edges that are not connected to the atoms (vertices) being compared, they are removed from the search space (for the bond-insensitive algorithm). Here we consider as *d-edges *only those edges that do not share similar bond types (for the bond-sensitive algorithm). Thus, by performing this initial pre-processing we limit the search space of the MCS. For example in the case of cyclopropane and isobutane the compatibility graph (Figure 1) will generate 12 vertices and 36 edges (18 of which are c-edges). With an increase in the number of bonds and atoms, the search space also increases due to the combination of edges and vertices. For example the compatibility graph between aqua(III)alamin (CAS:13422-52-1) and ferroheme B (CAS:14875-96-8) will generate 2,220 vertices with 2,706 c-edges and 1,526 d-edges, thus reducing the MCS search space. From the *c-edges*, we can obtain c-cliques using a modified Bron Kerbosch algorithm.

We have replaced the Koch [31] algorithm with an improved *c-cliques *algorithm for reporting unique cliques (i.e. removing cliques from the search space that had already been reported) proposed by Cazals and Karande [33,34]. We extended the algorithm to make it sensitive to bond type searches. A third improvement was introduced, which allowed only 10% of the reported d-edges (e.g. if the number of the *d-edges *are more than 20000 then only 2000 *d-edges *are selected) to find maximum c-cliques. This heuristic increases the speed of the algorithm by compromising on the size of the reported c-cliques, which are further extended by McGregor algorithm.

### Interplay Between CDKMCS and MCS+

The proposed algorithm in the SMSD checks if two molecules are identical or dissimilar based on the atom count and bond count before performing the MCS search. If the two molecules have identical atom count and bond count or their bond count and atom count are both dissimilar, then the native MCS module of the CDK (CDKMCS) is used. Else, the MCS+ algorithm is used. Hence the CDKMCS approach is used in cases where two molecules are identical or are complete subgraphs of each another. However, if the CDKMCS method is unable to find an optimal solution in the given time, the algorithm automatically uses the MCS+ method. This often occurs in cases of subgraphs with matching ring structures and dissimilar peripherals, *e.g*. ATP, ADP.

### VF+ library

VF algorithm is able to efficiently solve the graph isomorphism and subgraph isomorphism problems on Attributed Relational Graphs (ARG) [23,24]. It works effectively on very large graphs, since its memory requirements are smaller than those of other algorithms of the same kind. This makes it an obvious choice for handling larger graphs where the clique finding algorithms need more memory and time (such as CDKMCS and MCSPlus). Since this algorithm is very fast when graphs represent exact isomorphism or one is a subgraph of another, it is an ideal choice for substructure searches in cheminformatics. The Java code for VF algorithm for computing substructures was adapted from the MX library available at http://metamolecular.com/mx. The CDK compatible version of the code is available on http://wiki.github.com/asad/VFLib.

This algorithm also generates approximate MCS if two graphs are not isomorphic. Hence this becomes an ideal choice for big graphs where these approximate MCS solutions can further be extended *via *McGregor algorithm to find MCS. This makes it very efficient for finding MCS without compromising on the speed and memory usage. Therefore we further extended the original code to compute substructure search and MCS between two molecules.

### Introducing Chemical Knowledge into the MCS

The MCS search is based on two types of chemical constraints – a *bond-sensitive *MCS search and a *bond-insensitive *search. The bond sensitive module can be employed to screen compounds that mimic the substructure of the query molecule(s) based on the MCS. The bond-insensitive algorithm can be used for atom-atom mapping in a reaction where the focus is on bond changes, thus leading to a better understanding of the structural changes that emerge during a reaction. The runtime of the bond insensitive algorithm is longer than the bond-sensitive search as the former leads to an exponential increase in the search space. The SMSD software also contains several similarity (*i.e*. Tanimoto etc.) and distance descriptors (i.e. Euclidean etc.), which can be used as filters.

An MCS algorithm usually reports more than one solution when matching two molecules. Thus, even when the molecules are of identical size and chemical shape, there can be more than one feasible set of the MCS solutions (for example two benzene rings may have multiple MCS solutions but the solution can be filtered/constraint by chemical knowledge such as bond type).

We propose a chemically aware post-filtering (Figure 2) subroutine that deals with the ambiguous solutions arising from the MCS search. There are three filters applied:

a. Specific matching of the chemical functional groups, bond types (aromatic, non-aromatic, double, single etc.) and stereochemistry, e.g. phosphate, -SH, nitrite etc. are identified and matched.

b. The resulting solutions are sorted in ascending order of the total bond breaking energy (energy required to break the bonds between matched parts and unmatched parts) required by this MCS match (i.e. lowest energy is highest ranked).

c. The best set of solutions are chosen based on the above two steps and the solutions are then sorted in decreasing order according to the number of fragments generated if the matched part of the molecule is removed from the reference structure (i.e. if a three member ring is matched to a single ring structure then solutions which match the rings on the periphery are preferred over the central ring).

This leads to a set(s) of chemically relevant MCS solutions, keeping intact the chemical significance of the reported substructures.

### SMSD (Small Molecule Subgraph Detector)

SMSD is a combination of various algorithms (i.e. CDMCS, MCS+ and VF+ Lib). The decision to use an algorithm is purely based on the complexity of the input molecule. For example molecules, which potentially can be a subgraph based on atoms are handled by VF+ Lib first. Molecules whose bond count and atoms count are not equal are first matched by CDKMCS. If the solution is not computed within a limited time (i.e. timeout occurs) then it's passed to the MCS+, which starts the search from the scratch (i.e. solutions from CDKMCS is ignored). If the *d-edge *count is more than 99,999 then VF+ Lib is used to find MCS, as this is very efficient in handling medium and large sized graphs. McGregor further extends solution generated by VF+ Lib where possible to find MCS. A turbo-matching option was introduced in the SMSD, to return the first MCS match.

The MCS solution(s) are then passed to the chemical filters, which ranks the solutions in a chemically meaningful way. Thus we get chemically relevant MCS solutions computed in polynomial time.

## Results and Discussion

**Figure 4:**
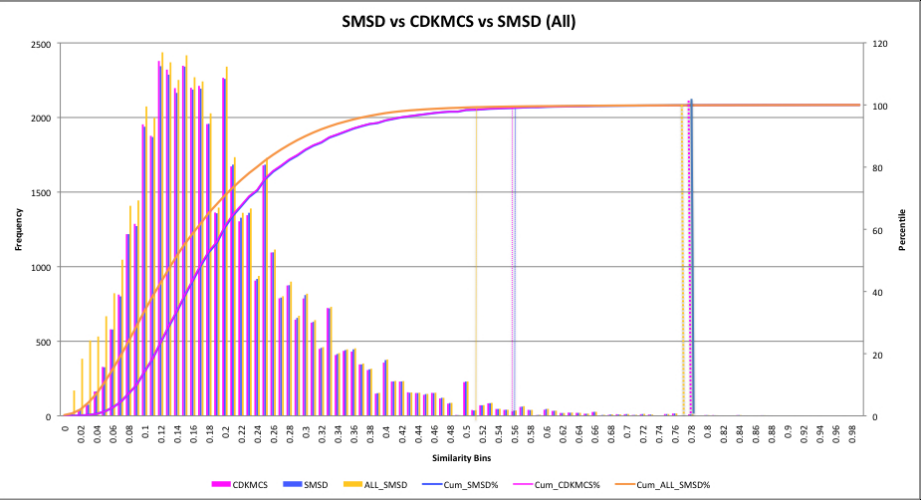
**Head to head comparison of MCS jobs processed by SMSD and CDK-MCS**. The similarity score frequencies (left axis) between each pair (colored boxes) of MCS solutions derived by the CDKMCS and the SMSD algorithm were sorted into bins ranging from 0 to 1 increasing in 0.01 increments. The cumulative percentage of the overall dataset for each bin is also shown (curves and right axis). Data shown in blue correspond to results from the SMSD algorithm while those in mauve correspond to CDKMCS. Data shown in yellow correspond to all the jobs that ran successfully by SMSD (includes 24 jobs which failed to run by CDKMCS). It is clear from the graph that the reported frequency of the SMSD similarity is almost similar to the CDKMCS similarity between the molecules. However the overall similarity between SMSD and CDKMCS is different because SMSD was able to process higher number of jobs than the latter. A good cut-off Tanimoto similarity score for reporting significant matches seems to be above 0.77 (at 99.9 percentile of the curve) for MCS based searches (indicated by the rightmost set of dashed lines).

The MCS in the SMSD was benchmarked (Figure 4) against the standard MCS module available in the CDK [30]. A random test set of 50,000 comparisons between PDB HET molecules [28] and KEGG ligands [27,35] was created. This list was filtered so that no comparison was repeated either with the same target and query, or *vice versa*. Thirty-eight examples in the test set did not find any similarity in either of the systems (CDKMCS and SMSD) due to unrecognised atom types, corresponding to heavy metal atoms in HET groups. This reduced the comparisons list to 49,962 jobs. The SMSD was able to compute all the jobs (except one which ran for more than an hour) while the native CDK-MCS failed to finish 24 jobs (~0.4%), mostly due to time-out or errors (jobs which ran more than 12 hours were automatically terminated by the machine).

### SMSD versus CDK similarity validation

A molecular fingerprint is derived by enumerating on various structural features of chemical space [1,36,37]. The rationale behind such an approach is to find "globally" analogous features between the query and target molecules under less stringent constraints than the MCS. There are several heuristic fragment based chemical similarity search approaches, like the 2-D fingerprint method, which represents chemical structure as a vector in a high-dimensional space. These approaches are usually very fast and good for the pre-screening of small molecules. One of the major limitations of such approaches is that they are unable to identify "local" similarity between structures thereby increasing the false-negative rates (i.e. identifying cases with weaker similarities).

**Figure 5:**
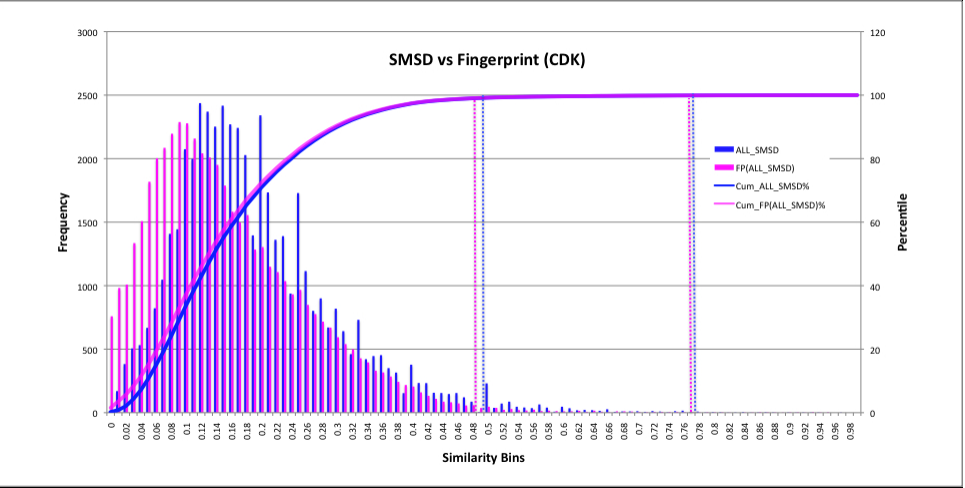
**The similarity score frequencies (left axis) between each pair (colored boxes) of MCS solutions derived by the CDK-Fingerprint and the SMSD algorithm were sorted into bins ranging from 0 to 1 increasing in 0.01 increments**. The cumulative percentage of the overall dataset for each bin is also shown (curves and right axis). Data shown in blue correspond to results from the SMSD algorithm while those in lilac correspond to CDK-Fingerprint. It is clear from the graph that the reported frequency of the SMSD similarity is different from the fingerprint similarity between the molecules. A good cut-off Tanimoto similarity score for reporting significant matches seems to be above 0.77 (at 99.9 percentile of the curve) for Fingerprint based searches and the MCS based search (indicated by the rightmost set of dashed lines).

In order to measure and benchmark validity (Figure 5) of the reported MCS solutions, we compared the SMSD results (49,962 jobs) with that of the Fingerprint similarity generated using the CDK fingerprint module. The results were compared to each other by binning according to the frequency of the similarity reported. The similarity scores between each pair of comparison molecules were sorted into bins. The cumulative percentage of the overall dataset for each bin was also calculated (Figure 5). A graph-matching score defined as *c*/(*a *+ *b *- *c*) where c is the size of the MCS and a, b are number of atoms in query and target respectively. A score of 0.49–0.50 was found to represent a good cut-off to use for graph matching using the SMSD, as 99% of all random matches occur below this threshold. Likewise, a similarity score of ~0.77 represents a good cut-off to use the SMSD and fingerprint matching using the CDK Fingerprints, as 99.9% of all random matches occur below this threshold. These cut-off scores (similarity) can be used to filter out molecules [38] which have a very high probability of being dissimilar. This analysis also highlights the fact that the similarity range covered by fingerprints is very different to that of the MCS search as reported earlier [39].

### SMSD versus CDKMCS similarity

A second set of benchmarks (Figure 4) was performed between the CDKMCS and the SMSD on the subset of intersection (45,139 jobs that reported at least one match between query and target molecules) between successful cases reported by both the methods. A detailed analysis shows that in 43,711 (~96.83%) cases, the SMSD and the CDKMCS searches reported an MCS of similar size. In 1,361 (~3.01%) cases the SMSD reported an MCS of a longer length than the MCS reported by the CDKMCS and in only 67 (~0.14%) instances was it vice versa. The difference in the size of the matches is not due to time out. If a time out occurs in CDKMCS then the MCS+ starts to compute MCS from the beginning. Previously, the CDKMCS used to provide better matches (though very expensive in terms of time and memory) than MCS+, in a few cases. This was due to the fact that these molecules had a very high *d-edge *count; hence MCS+ use to switch into heuristic mode. We have now been able to resolve this issue using VF+ Lib, which can handle larger graphs efficiently. The analysis presented in this paper is performed without VF+ Lib so as to highlight the strength of the combination of MCS+ and CDKMCS. Some larger matches reported by CDKMCS are incorrect due to the MCIS vs MCES problem. For example in the case of cyclopropane and isobutene, CDKMCS reports 3 matched atoms whereas MCS+ reports only 2, which is correct. This highlights the strength of the SMSD and its ability to calculate the MCS in a reasonably acceptable time without clogging the comparison pipeline.

### The MCS Execution Runtime

All these test cases were run on EBI farm nodes (processor speed varies across the nodes) with an average of 300 jobs processed in parallel. The overall execution time for all the processed jobs can be obtained by summing up the CPU time. This time does not have a direct, one to one correlation between the run times of jobs (e.g. Job "A" fired from the SMSD ran on a slower machine whereas same job "A" fired by the CDKMCS ran on a faster machine or vice versa). Hence this is just an indicative measure of the runtime performance between the algorithms.

The total time required by a single processor to run 49,961 test cases using the SMSD was ~21 hours whereas 49,938 test cases processed by the CDKMCS, took ~113 hours (excluding 25 jobs which were still running even after 6 hours). Hence the average execution time (in the completed job list) for each comparison by the SMSD is less than ~1.5 seconds and ~8.2 seconds by the CDKMCS. Thus overall, execution of the SMSD is far faster than the CDKMCS.

## Conclusion

MCS have been previously applied to identify biochemical activity in the metabolic pathways by mapping atom-atom equivalences between reactants and products in a chemical reaction [15,40-43]. The algorithms used for screening similar molecules are usually bond sensitive (i.e. discriminate between single and double bonds) whereas MCS similarity checks between reactant and product molecules in a reaction are often bond insensitive (i.e. atom-atom mapping is preferred over bond type matches). We propose the SMSD as a robust software for calculating substructure similarity using the MCS algorithm. The SMSD returns more MCS solutions than CDKMCS, as in many cases the latter fails to detect similarity due to timeout. The SMSD software encapsulates the power of various algorithms and generates chemically relevant MCS solutions. Another merit of this software is that the MCS solutions are ranked on the basis of their chemical relevance such as bond energy, fragment size etc. This is one of the first attempts at ranking the MCS solutions in a chemically meaningful manner.

Presently this tool can be applied to various areas of bioinformatics and chemo-informatics for finding exhaustive MCS matches. For example, it can be used to analyse metabolic networks by mapping the atoms between reactants and products involved in a reaction. It can also be used to detect the MCS/substructure searches in small molecules reported by metabolome experiments, as well as in the screening of drug-like compounds with similar substructures.

This can be used to determine whether or not a query was a substructure of the target, as this question does not require an exhaustive MCS match with all possible maximum cliques.

## Availability and requirements

Project name: Small Molecule Subgraph Detector (SMSD)

Project home page: http://www.ebi.ac.uk/thornton-srv/software/SMSD/

Operating system(s): Platform independent (Windows, MAC, Linux/Unix)

Programming language: Java

Other requirements: Java 1.6 or higher

License: Creative Commons http://creativecommons.org/licenses/by/3.0/ 

Any restrictions to use by non-academics: NONE

## Abbreviations

MCS: Maximum Common Subgraph; CDK: Chemistry Development Kit; PDB: Protein Data Bank; KEGG: Kyoto Encyclopedia of Genes and Genomes; CDKMCS: MCS in the Chemistry Development Kit; SMSD: Small Molecule Subgraph Detector; MCS+: Maximum Common Subgraph Plus; MCIS: Maximum Common Induced Subgraph; MCES: Maximum Common Edge Subgraph; *G*_*m*_: Molecular graph; *G*_*q*_: Query graph; *G*_*t*_: Target graph.

## Competing interests

The authors declare that they have no competing interests.

## Authors' contributions

SAR was involved in designing the algorithm, coding the software and manuscript preparation. He was also involved in testing and benchmarking the tool. MB was involved in testing and benchmarking the tool. GLH was involved in testing the chemical relevance of the tool and in the process of the manuscript preparation. RS was involved in the supervision of the algorithm development. JMT was involved in the overall supervision of the project, manuscript preparation, intellectual inputs and guidance.

## Authors' informations

**SAR **is a Research Fellow in the JMT group.

**MB **is a Research Fellow in the JMT group.

**GLH **is a Research Fellow in the JMT group.

**RS **is a full time Professor at ZAIK, University of Cologne, Germany.

**JMT **is the Director, Research Programme Coordinator and Group Leader at EMBL-EBI, Cambridge, UK. She has been a Fellow of The Royal Society since 1999.

## Appendix 1: SMSD Algorithm for Calculating MCS

**PROCEDURE SMSD**

   INPUT: Given a query Gq and a target Gt graph

   OUTPUT: the MCS mappings between the two graphs

   IF atoms of query Gq is a subset of Gt or *vice versa*

      THEN CALL VF+ (Gq, Gt)

   ELSE IF the Gq bond count NOT EQUAL TO Gt && Gq bond

      count NOT EQUAL TO Gt

      THEN CALL CDK+ (Gq, Gt)

      IF CDKMCS (Gq, Gt) EQUALS TO NULL

         THEN CALL MCS+ (Gq, Gt)

      END IF

      IF MCS+ (Gq, Gt) EQUALS TO NULL

         THEN CALL VF+ (Gq, Gt)

      END IF

   ELSE

      CALL VF+ (Gq, Gt)

      END IF

   END IF

**END PROCEDURE**

**PROCEDURE VF+ Lib**

   INPUT: Given a query Gq and a target Gt graph

   OUTPUT: the mappings between the two graphs

   IF node count of Gq > node count Gt

      THEN CALL VF+ (Gt, Gq)

   ELSE CALL VF+ (Gq, Gt)

   END IF

   IF the MCS size EQUALS Gq node count OR

      MCS Size EQUALS Gt node Count

      THEN return MCS

   ELSE CALL McGregor (Gq, Gt, MCS)

   END IF

**END PROCEDURE**

**PROCEDURE MCS+**

   INPUT: Given a query Gq and a target Gt graph

   OUTPUT: the mappings between the two graphs

   Construct compatibility graph and mark c-edges and d-edges

   IF heuristic flag False and d-edge count > 99,999

      THEN return NULL

   ELSE

      Choose only 10% of the reported *d-edges*

   ENDIF

   CALL CazalsKarandeKoch (CompatibilityGraph, c-edges, d-edges)

   IF the MCS size EQUALS Gq node count OR

      MCS Size EQUALS Gt node Count THEN return MCS

   ELSE CALL McGregor (Gq, Gt, MCS)

   END IF

**END PROCEDURE**

**PROCEDURE CDKMCS**

   INPUT: Given a query Gq and a target Gt graph

   OUTPUT: the mappings between the two graphs

   IF node count of Gq > node count Gt

      THEN CALL UniversalIsomorphismTester (Gt, Gq)

   ELSE CALL UniversalIsomorphismTester (Gq, Gt)

   END IF

   IF timeout is TRUE THEN return NULL

   ELSE return MCS

**END PROCEDURE**
